# Supplementary figures and images for: Morphometric Relationships between Length and Weight of 109 Fish Species in the Caribbean Sea (French West Indies)
Source: Animals (Basel). 2023 Dec 14;13(24):3852. doi: 10.3390/ani13243852 (PMC10740606; doi:10.3390/ani13243852)

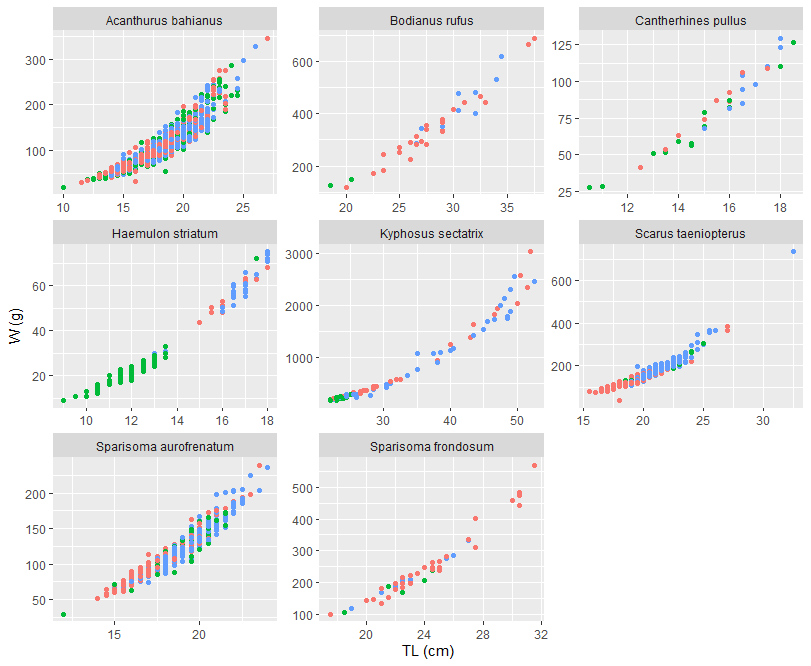

Supplement: Supplementary file 1 [file animals-13-03852-s001.zip › Figure S1.tiff]
